# A112 THE EDMONTON PANCREATICOBILIARY INFLAMMATION AND CANCER (EPIC) PROGRAM – A NEW MULTIDISCIPLINARY COORDINATION OF CARE INITIATIVE

**DOI:** 10.1093/jcag/gwad061.112

**Published:** 2024-02-14

**Authors:** G S Sandha, H Abdulhussain, T Krahn, M Timmermans, G Buchanan, E Bereznicki, P Mathura, A Kwan, S Perryman, S Veldhuyzen van Zanten

**Affiliations:** University of Alberta College of Health Sciences, Edmonton, AB, Canada; University of Alberta College of Health Sciences, Edmonton, AB, Canada; University of Alberta College of Health Sciences, Edmonton, AB, Canada; Alberta Health Services, Edmonton, AB, Canada; Alberta Health Services, Edmonton, AB, Canada; Alberta Health Services, Edmonton, AB, Canada; Alberta Health Services, Edmonton, AB, Canada; Alberta Health Services, Edmonton, AB, Canada; Alberta Health Services, Edmonton, AB, Canada; University of Alberta College of Health Sciences, Edmonton, AB, Canada

## Abstract

**Background:**

Pancreaticobiliary (PB) cancer is associated with a poor prognosis. A timely diagnosis and a coordinated, multidisciplinary effort may improve outcomes as patients navigate the healthcare system for investigations and treatment.

**Aims:**

To establish and pilot a multidisciplinary (gastroenterology [GI], hepatopancreatic biliary [HPB] surgery, radiology, oncology, palliative medicine, and nutrition) collaborative care pathway (CCP) to coordinate timely referral and access to management for patients with PB cancer.

**Methods:**

With multidisciplinary expert input, a CCP for PB cancer patients was developed. A donation to our hospital foundation facilitated the hiring of a nurse navigator (NN) to coordinate this process. The EPIC program was initiated as a 6-month pilot project on 01/07/23 for all consecutive patients referred to the University of Alberta Hospital with suspected PB cancer. Referrals were triaged by GI and HPB surgery into resectable, borderline resectable, or unresectable arms of the CCP. EUS biopsy and/or ERCP for stent were performed as needed. A referral to medical oncology was made after a diagnosis was established. Descriptive statistics were completed.

**Results:**

At 3 months, 64 patients (39 M, 25 F), mean age 67±12 years (range 19-95 years), were referred to EPIC. Presenting symptoms were abdominal pain (73%), weight loss (40%), jaundice (27%), nausea/vomiting (21%), itching (8%), and new-onset diabetes (5%). Site of cancer was pancreas (77%, 49/64), bile duct (20%, 13/64), and ampulla (3%, 2/64). Timelines to care access were compared with a similar pre-CCP patient cohort as shown in Table.

**Conclusions:**

The role of the NN is to reduce variability and coordinate timely referral, scheduling, and follow-up based on the care pathway, and to serve as a point of contact for ongoing patient issues. The EPIC program pilot is on track to meet the timelines set forth in the care pathway. Primary care outreach to increase awareness of symptoms and for prompt investigation may further improve access to care in these patients.

Note: negative numbers indicate days where the GI/HPB review occurred before the CT/MRI, or intervention before GI/HPB review

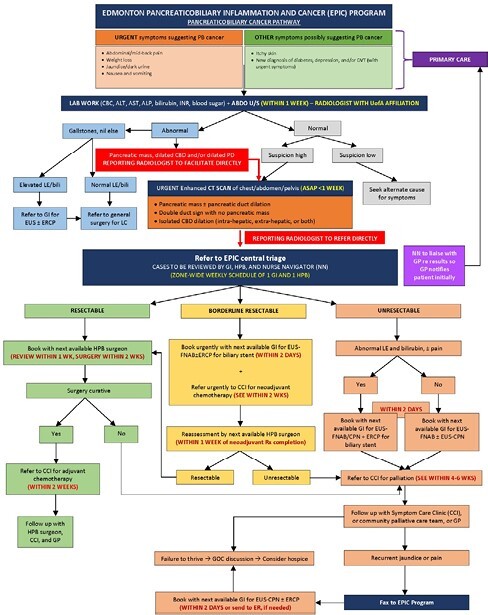

EPIC COLLABORATIVE CARE PATHWAY

**Funding Agencies:**

None

**Table:** 

TIMELINES (Days, mean±SD [range])	PRE-CCP (n=69)	Post-CCP (n=64)
ONSET OF SYMPTOMS→CT/MRI	40±13 [0, 366]	74±93 [1, 391]
CT/MRI→EPIC REVIEW (GI/HPB)	7±16 [-33, 53]	7±10 [-8, 35]
EPIC REVIEW (GI/HPB)→INTERVENTION (EUS/ERCP)	7±14 [0, 60]	4±8 [-27, 27]
INTERVENTION (EUS/ERCP)→SURGERY	29±19 [5, 61]	23±17 [2, 47]
CT/MRI→SURGERY	31±18 [5, 61]	34±13 [16, 53]
REFERRAL TO ONCOLOGY→SEEN BY ONCOLOGIST	35±21 [0, 113]	35±10 [9, 48]

